# Neuronal nitric oxide synthase expressing neurons: a journey from birth to neuronal circuits

**DOI:** 10.3389/fncir.2012.00082

**Published:** 2012-12-05

**Authors:** Ludovic Tricoire, Tania Vitalis

**Affiliations:** ^1^CNRS-UMR 7102, Laboratoire de Neurobiologie des Processus Adaptatifs, Université Pierre et Marie CurieParis, France; ^2^CNRS-UMR 7637, Laboratoire de Neurobiologie, ESPCI ParisTechParis, France

**Keywords:** interneurons, GABA, development, nNOS, specification, classification

## Abstract

Nitric oxide (NO) is an important signaling molecule crucial for many physiological processes such as synaptic plasticity, vasomotricity, and inflammation. Neuronal nitric oxide synthase (nNOS) is the enzyme responsible for the synthesis of NO by neurons. In the juvenile and mature hippocampus and neocortex nNOS is primarily expressed by subpopulations of GABAergic interneurons. Over the past two decades, many advances have been achieved in the characterization of neocortical and hippocampal nNOS expressing neurons. In this review, we summarize past and present studies that have characterized the electrophysiological, morphological, molecular, and synaptic properties of these neurons. We also discuss recent studies that have shed light on the developmental origins and specification of GABAergic neurons with specific attention to neocortical and hippocampal nNOS expressing GABAergic neurons. Finally, we summarize the roles of NO and nNOS-expressing inhibitory neurons.

## Introduction

Information processing within neocortical and hippocampal circuits relies upon complex interactions between glutamatergic excitatory projection neurons and GABAergic inhibitory neurons. Coordinated cell–cell communication amongst and between these two neuronal populations is essential to maintain a delicate balance between excitatory and inhibitory signaling within the brain and is subject to dynamic regulation by many neuromodulatory substances such as various neuropeptides and nitric oxide (NO) (Krimer and Goldman-Rakic, 2001; Baraban and Tallent, 2004; Somogyi and Klausberger, 2005). Disruption of this excitatory-inhibitory balance often precipitates pathological disorders such as epilepsy, autism, and schizophrenia (McBain and Fisahn, 2001; Rubenstein and Merzenich, 2003; Levitt et al., 2004; Batista-Brito et al., 2009; Lewis et al., 2011; Marin, 2012). Understanding normal brain functions and the bases of these pathologies requires thorough characterization of telencephalic neurons and their development. For GABAergic neurons this has proven particularly difficult due to their remarkable diversity. Indeed a prerequisite in determining the circuit properties of this cell group is to first define each specific class of interneuron that populates the telencephalon. Helpful criteria for such classification were recently established by the Petilla inteneuron nomenclature group (PING). These include morphological, electrophysiological and molecular properties (Petilla Interneuron Nomenclature Group et al., 2008). Among the established subtypes of interneurons the subpopulation expressing neuronal nitric oxide synthase (nNOS) was recently shown to represent the most prevalent interneuron subpopulation in the hippocampus (Fuentealba et al., 2008). Though historically these cells had received relatively little attention a wave of recent studies have implicated interneurons expressing nNOS in important physiological processes such as the homeostatic regulation of sleep (Kilduff et al., 2011), neurovascular coupling to control neocortical blood flow (Cauli et al., 2004; Cauli and Hamel, 2010; Perrenoud et al., 2012b in this issue), and synaptic integration of adult born neurons (Overstreet and Westbrook, 2003). Moreover, these interneurons may contribute to pathological states related to dysfuntion of NO production/release as has been documented in neuronal death and epilepsy (Gholipour et al., 2010). Despite the common expression of nNOS there exists considerable heterogeneity within this cohort of interneurons yielding even further subdivision and overlap with other subpopulations defined by criteria unrelated to nNOS expression. During the past decade studies focusing on the developmental origins (place and date of birth) and genetic programs underlying fate specification have produced additional criteria that help make sense of interneuron diversity. In this review we will summarize recent advances in the characterization of neocortical and hippocampal nNOS expressing interneurons with particular emphasis on the genetic programs governing their genesis and specification. We will also briefly review the current understanding of circuit roles played by interneurons expressing nNOS in the development and plasticity of the hippocampus and neocortex.

## GABAergic neurons expressing neuronal nitric oxide synthase in the juvenile or mature hippocampus and neocortex

Using a combination of intracellular recoding, dye filling, single cell RT-PCR, NADPH-diaphorase (NADPH-d) reactivity and immunostaining with various antibodies against calcium binding proteins, neuropeptides and nNOS, several groups have shown that nNOS GABAergic neurons can be subdivided into several hippocampal and neocortical sub-populations that are summarized in Tables 1 and 2 and Figure 3 (see below).

**Table 1:** **Characteristics of rodent hippocampal GABAergic neurons expressing neuronal nitric oxide synthase**.

**Markers**	**Morphology location**	**Axonal targeting on pyramidal neurons**	Firing pattern^*^	**Transcription factors or lineage markers**	**Place of genesis^£^**
nNOS^+^/NPY^+^	Multipolar^1^^,^^2^^,^^3^	Dendrite^1^^,^^2^^,^^3^	Late spiking^3^	Nkx2.1/Lhx6^3^^,^^4^	MGE^3^^,^^4^^,^^5^
(IVCs)	s.r.; s.p.; s.o.		Non-adapting		AEP/POA?
nNOS^+^/NPY^+^	Multipolar^3^^,^^6^^,^^7^	Dentritic shaft^6^^,^^7^	Late spiking^3^^,^^5^^,^^6^	Nkx2.1/Lhx6^3^^,^^4^	MGE^3^^,^^4^^,^^5^
(NGFCs)	neurogliaform	Blood vessels	Non-adapting	CoupTFII	AEP/POA ?
	s.l.m./s.r. bd				
	s.l.m./s.m. bd				
nNOS^+^/VIP^+^/CR^+^	Bipolar^8^ s.p.	SOM^+^ neurons^8^ of the s.o.	Non-LS^3^	CoupTFII^4^	CGE^3^^,^^4^
				5-HT_3A_^4^	LGE ?
					AEP/POA ?
nNOS^+^/PV^+^	Basket?	Granule cell layer?	Fast spiking?	Nkx2.1?	MGE?
	DG specific				

* Firing pattern elicited from intracellular injections of depolarizing currents.

£ AEP, entopeduncular area; CGE, caudal ganglionic eminence; LGE, lateral ganglionic eminence; MGE, medial ganglionic eminence; POA, preoptic area; s.l.m.; stratum lacunosum molecular; s.m.; startum molecular; s.o.; stratum oriens; s.p.; stratum pyramidale; s.r.; stratum radiatum.

1 *Fuentealba et al., 2008*,

2 *Somogyi et al., 2012*,

3 *Tricoire et al., 2010*,

4 *Tricoire et al., 2011*,

5 *Jaglin et al., 2012*,

6 *Price et al., 2005*,

7 *Zsiros and Maccaferri, 2005*,

8 Freund and Buzsáki, 1996.

**Table 2:** **Characteristics of rodent neocortical GABAergic neurons expressing neuronal nitric oxide synthase**.

**Markers**	**% cells within nNOS-type II**	**Morphology**	**Axonal targeting**	Firing pattern^*^	**Transcription factors or lineage markers**	**Place of genesis**
Highly		Long projection^1^^,^^2^	Blood vessels^1^^,^^2^	Late spiking^3^^,^^8^	Nkx2.1/Lhx6^3^^,^^4^^,^^5^	MGE^3^^,^^4^^,^^5^
nNOS^+^SOM^+^/NPY^+^^3^^,^^4^^,^^5^^,^^6^^,^^7^^,^^8^			Neurons^1^^,^^2^	Adapting^3^^,^^8^		
(nNOS-type I)						
Lightly nNOS^+^	55	Neurogliaform^3^^,^^6^	Blood vessels ?	Adapting^3^^,^^8^	Nkx2.1/Lhx6^3^^,^^4^	MGE^3^^,^^4^
NPY^+^^3^^,^^5^^,^^6^^,^^8^^,^^9^			Dentritic shaft		5-HT_3A_^3^^,^^4^^,^^6^	CGE/AEP^3^^,^^4^
(nNOS-type II)						
Lightly nNOS^+^ PV^+^ or SOM^+^^3^^,^^5^^,^^6^^,^^8^^,^^10^ (nNOS-type II)	35	Multipolar^3^	Blood vessels ? Proximal dendrites Soma Axonal initial segment	Fast spiking^3^	Nkx2.1/Lhx6^3^^,^^4^	MGE^3^^,^^4^
Lightly nNOS	10	Bipolar^3^^,^^6^	Blood vessels ?	Adapting^3^	5-HT_3A_^3^^,^^4^^,^^6^	AEP/PO? ^3^
VIP^+^/CR^+^^3^^,^^5^^,^^6^^,^^8^		Double-bouquet^3^^,^^6^	Soma			CGE^3^^,^^4^
(nNOS-type II)						SVZ/Ctx?

* Firing pattern elicited from intracellular injections of depolarizing currents: AEP, entopeduncular area; CGE, caudal ganglionic eminence; Ctx, cortex; LGE, lateral ganglionic eminence; MGE, medial ganglionic eminence; POA, preoptic area; SVZ, subventricular zone.

1 *Tomioka et al., 2005*,

2 *Higo et al., 2009*,

3 *Perrenoud et al., 2012a*,

4 *Magno et al., 2012*,

5 *Jaglin et al., 2012*,

6 *Perrenoud et al., 2012b*,

7 *Kubota et al., 2011*,

8 *Karagiannis et al., 2009*,

9 *Oláh et al., 2009*,

10 Vruwink et al., 2001.

### GABAergic neurons expressing neuronal nitric oxide synthase in the hippocampus

The hippocampus is subdivided in two main anatomical areas, the dentate gyrus (DG) and the cornu ammonis (CA). The CA region is classically further divided into CA1–4. In this section of the review, we will mainly focus on results obtained in CA1 where interneuron diversity has been best characterized but will detail other areas when data are available. As in the neocortex, nNOS expressing neurons comprise primarily inhibitory GABAergic neurons although nNOS immunoreactivity is also found in CA1 pyramidal cells. In these glutamatergic excitatory cells staining intensity in mature brain is much weaker than in interneurons and nNOS is observed preferentially in dendritic spines (Burette et al., 2002). Hippocampal nNOS expressing interneurons differ from their neocortical homologs in that they are much more abundant and the level of nNOS expression is more homogenous (Jinno and Kosaka, 2002). Indeed, while neocortical nNOS^+^ interneurons may be subdivided based on intensity of nNOS immunoreactivity (see next section), no such distinction exists in the hippocampus. Furthermore, a recent study revealed that interneurons expressing nNOS comprise the most abundant interneuron subpopulation in the hippocampus, in contrast to neocortical observations where parvalbumin (PV) expressing interneurons are considered to be the most abundant interneuron subtypes (Fuentealba et al., 2008). Like in the neocortex, nNOS expressing interneurons are found in all hippocampal layers of CA and in the DG. One study in the mouse has shown that the density of nNOS interneurons is higher in the septal/dorsal part compared to the temporal/ventral part of the hippocampus (Jinno and Kosaka, 2002).

**Figure 1:**
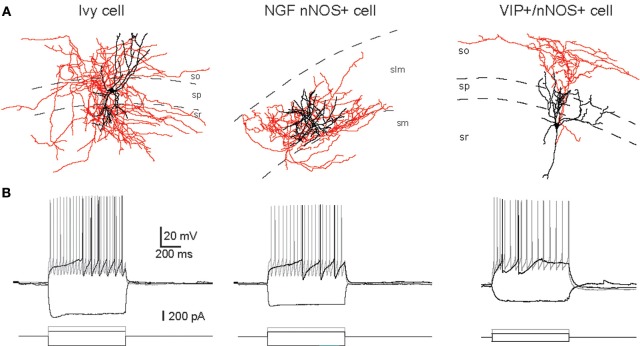
**Examples of IvC, NGFC, and VIP^+^/nNOS^+^ interneurons. (A)** Neurolucida reconstructions of biocytin-filled cells (black, dendrite; red, axon). **(B)** Voltage responses of cells shown in **(A)** to three current step injections (-200 pA, just suprathreshold, and twice the current for just suprathreshold). Adapted from Tricoire et al. (2010).

In rats and mice, at least five interneuron subpopulations have been described to express nNOS: (1) the neurogliaform cells (NGFC), (2) Ivy cells (IvC), (3) interneurons co-expressing the vasoactive intestinal peptide (VIP) and calretinin (CR), (4) interneurons expressing PV and (5) projection cells. This latter subtype of nNOS^+^ cells has been shown to accumulate close to the subiculum (Freund and Buzsáki, 1996). The subpopulation coexpressing nNOS and PV principally resides in the DG (Dun et al., 1994; Jinno and Kosaka, 2002, 2004). However species differences between rat and mouse have been noted as co-expression of nNOS and PV in rat DG is much lower than in mouse (Dun et al., 1994 for rat; Jinno and Kosaka, 2002 for mouse). Additionally, a subset of somatostatin (SOM) expressing interneurons in CA1, CA3, and DG areas has been shown to express nNOS (Jinno and Kosaka, 2004). Similar to the case with PV, species differences have been encountered with nNOS/SOM coexpression being higher in rat than mouse (Dun et al., 1994 for rat; Jinno and Kosaka, 2004 for mouse). Examples of the morphology and firings of three of these cell groups are provided in Figure 1.

### Neurogliaform and ivy cells

Hippocampal NGFCs derive their name from their neocortical homologs with which they share common morphological features. NGFC bodies are typically found in stratum lacunosum moleculare (slm) and its border with s. radiatum (sr) of CA1-3, as well as within s. moleculare of the DG (Vida et al., 1998; Price et al., 2005, 2008; Elfant et al., 2008; Karayannis et al., 2010; Szabadics et al., 2010; Armstrong et al., 2011; Krook-Magnuson et al., 2011; Markwardt et al., 2011). Their soma is relatively small in comparison with those of other interneuron subtypes such as somatostatin^+^ (SOM^+^) and PV^+^ interneurons. NGFCs exhibit a multipolar dendritic network with a high degree of ramification close to the soma without any privileged orientation. The axonal arborization is extremely dense with extensive ramification within the local network and usually radiates beyond the spatial boundaries of the dendritic field (Price et al., 2005; Tricoire et al., 2010). In addition, both fields are restricted to slm and typically penetrate very little into the sr. However, several studies reported that the axons of CA1 NGFCs may penetrate s. moleculare of the DG (Price et al., 2005; Fuentealba et al., 2010; Tricoire et al., 2010). Similarly axons of DG NGFCs can cross the hippocampal fissure and penetrate into slm of nearby CA1 and subiculum (Armstrong et al., 2011).

Closely related to NGFCs, are the recently described hippocampal IvCs (Fuentealba et al., 2008, 2010; Tricoire et al., 2010, 2011; Krook-Magnuson et al., 2011) and the existence of an equivalent interneuron subpopulation in the neocortex is a matter of debate. These cells were first reported by Peter Somogyi's group and named for the English Ivy-like appearance of their axons which profusely branch close to their origin providing dense thin branches with numerous small varicosities (Fuentealba et al., 2008; Somogyi et al., 2012 in this issue). In contrast to NGFCs, the cell bodies and processes of IvCs are found in s. oriens, s. pyramidale and sr without infiltrating slm (Fuentealba et al., 2008; Tricoire et al., 2010). However, recent results indicate that IvCs whose soma is located in sr regularly send axons and dendrites to some extent in slm. (Somogyi et al., 2012 in this special issue and Szabo et al., 2012).

From a molecular point of view, NGFCs and IvCs express several common markers/receptors resulting in convergent neurochemical profiles for these two nNOS^+^ interneurons subtypes. The neuropeptide Y (NPY) has been found to colocalize with nNOS in both NGFCs and IvCs (Fuentealba et al., 2008; Tricoire et al., 2010; Somogyi et al., 2012 in this issue). However, NPY is not specific to nNOS^+^ interneurons as it is also frequently coexpressed with SOM and PV in yet other distinct interneuron subpopulations (Klausberger and Somogyi, 2008). Whereas IvC and NGFC subpopulations of CA constitute a distinct population from PV and SOM expressing subpopulation, nNOS and PV often colocalize in the DG. The alpha1 GABAA receptor subunit is also frequently encountered in IvCs and nNOS^+^ NGFCs (Fuentealba et al., 2008; Tricoire et al., 2010) but, like NPY, it cannot be considered as a specific marker of IvCs or NGFCs as it is also expressed in other interneuron subtypes (Baude et al., 2007). More recently, the delta GABAA receptor subunit that underlies tonic inhibition was demonstrated to preferentially localize to NGFC/IvC interneurons (Oláh et al., 2009). However this subunit is not specific of interneurons and is also found in excitatory granule cells in DG (Wei et al., 2003). IvCs and NGFCs are inhibited by mu opioid agonists, such as DAMGO, consistent with the expression of mu opioid receptors (MORs) on both interneuron subpopulations (Krook-Magnuson et al., 2011). Interestingly, MORs are also found in PV^+^ interneurons in CA1. This expression pattern is distinct from that observed in neocortex where MORs are found on interneurons co-expressing VIP and cholecystokinin (CCK) (Férézou et al., 2007). The microtubule associated protein alpha actinin 2 has been shown to be selective for NGFCs and IvCs in rat hippocampus (Price et al., 2005; Fuentealba et al., 2008). It is not clear if it is also the case in mouse hippocampus. In rat, the chicken ovalbumin upstream promoter transcription factor II (CoupTFII) is frequently observed in both IvCs and NGFCs (Fuentealba et al., 2010), whereas in mouse it is rarely found in IvCs despite frequent expression in NGFCs (Tricoire et al., 2010). So far reelin appears to be the only marker that is differentially expressed between IvCs and NGFCs although this marker is also commonly found in SOM^+^ interneurons (Alcántara et al., 1999). Indeed, reelin has been detected in NGFCs but not in IvCs (Fuentealba et al., 2010; Somogyi et al., 2012 in this issue).

In CA1, IvCs receive their main excitatory inputs from CA1 and CA3 pyramidal cells (Fuentealba et al., 2008; Somogyi et al., 2012 in this issue) while NGFCs receive excitatory inputs from the entorhinal cortex via the temporo-ammonic pathway and from CA3 via the Schaffer collateral pathway (Price et al., 2005). Both cell subpopulations inhibit down-stream targets via GABAA receptors. However, in addition, NGFCs generate long lasting postsynaptic inhibitory currents through the activation of GABAB receptors on their postsynaptic targets (Price et al., 2005, 2008). Interestingly, NGFCs are highly interconnected via both electrical and chemical synapses (Price et al., 2005; Zsiros and Maccaferri, 2005). In contrast, IvCs have thus far only been found to signal via chemical synapses on postsynaptic cells (Fuentealba et al., 2008). In terms of neuronal activity, IvCs and NGFCs exhibit very similar electrophysiological properties regarding their passive membrane and firing properties (Tricoire et al., 2010). For example, they all show a late spiking phenotype, i.e., a delay to generate action potentials when challenged by just suprathreshold current injection (Price et al., 2005; Zsiros and Maccaferri, 2005; Tricoire et al., 2010). None of these cell types exhibit adaptation of firing frequency at threshold stimulation. However, upon stronger stimulation, they all switch to an adaptive spiking profile (Tricoire et al., 2010). Nonetheless, *in vivo* recordings in anesthetized rats revealed that IvCs and NGFCs exhibit different firing characteristics during rhythmic hippocampal activities. NGFCs fire at the peak of theta oscillations detected extracellularly in s. pyramidale, whereas IvCs fire at the trough of these oscillations (Fuentealba et al., 2010; Lapray et al., 2012).

### VIP^+^/CR^+^/nNOS^+^ interneurons in CA1-3

The third interneuron subpopulation expressing nNOS consists of a subset of VIP^+^/CR^+^ interneurons (Jinno and Kosaka, 2002; Tricoire et al., 2010). This population is specialized to innervate other GABAergic cells exclusively. To date, three types of interneuron-specific (IS) interneurons have been described on the basis of their anatomical and neurochemical features (Acsády et al., 1996a,b; Gulyás et al., 1996). Among them, nNOS has been found in the IS-3 subset (Tricoire et al., 2010). These cells have somas located in stratum pyramidale (s.p.) or in stratum radiatum (s.r.) close to the pyramidal layer, dendritic fields that are vertically oriented, and a primary axon descending to emit several horizontally oriented branches at the s.o.-alveus border. Consistent with their axonal morphology, they constitute a major local source of inhibition to SOM^+^ O–LM cells (Acsády et al., 1996a,b; Gulyás et al., 1996; Chamberland et al., 2010). Electrophysiologically, they exhibit an irregular firing pattern when depolarized with current injection which differs from the late spiking and more regular firing profile of IvC/NGFC (Tricoire et al., 2010). The position of these neurons in the hippocampal network in terms of input is still to be determined.

### PV^+^/nNOS^+^ interneurons in DG

The expression pattern of nNOS in the DG differs from that observed in CA areas. Indeed, nNOS is found in about 20% of PV^+^ interneurons (Jinno and Kosaka, 2002) whereas there was no overlap between nNOS and PV expression in CA areas. While PV^+^ interneurons in DG are well characterized in terms of morphology and neurophysiology (Bartos et al., 2007), so far no study has examined if nNOS^+^/PV^+^ cells represent a specific interneuron subpopulation compared to other DG PV^+^ interneurons. Briefly, PV^+^ interneurons exhibit a fast spiking firing profile, which means that they are able to generate a train of action potentials at high frequency and little to no accommodation when injected with depolarizing current. Action potentials in these neurons are much shorter in duration that those in IvC/NGF (Tricoire et al., 2011) and their axons preferentially target the perisomatic region of granule cells making them ideally suited to rapidly regulate DG output.

### GABAergic neurons expressing neuronal nitric oxide synthase in the neocortex

**Figure 2:**
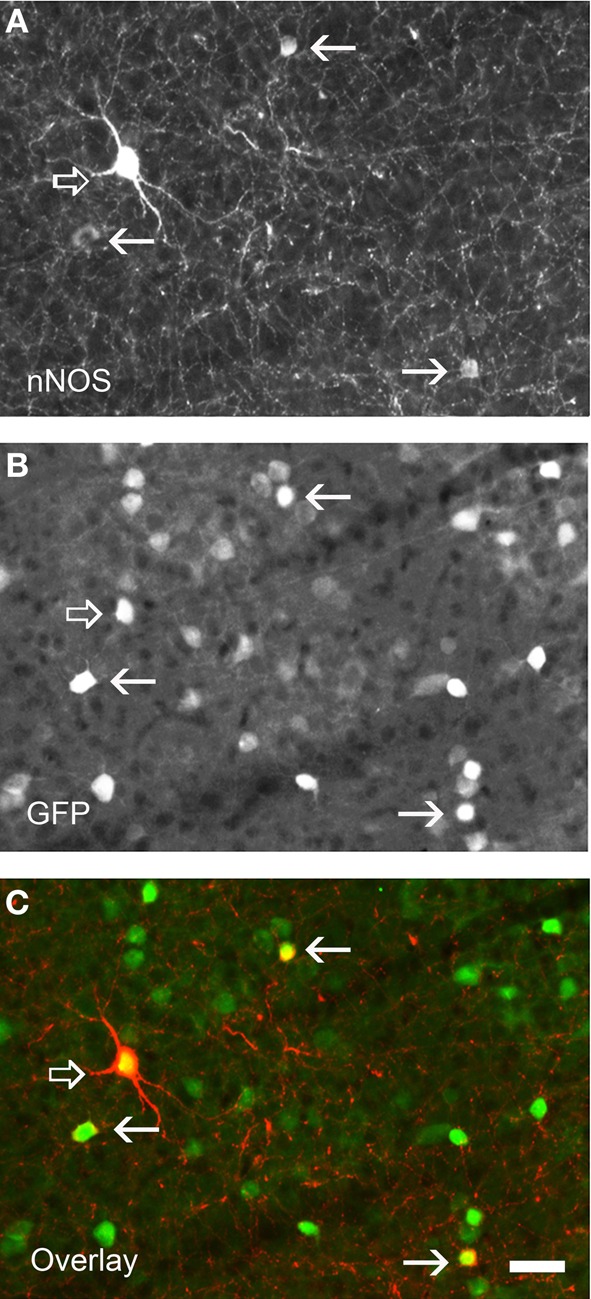
**Immunolabeling for nNOS in a neocortical sections of GAD67:GFP mouse strain showing the two nNOS populations. (A)** Fluorescence picture showing immunohistochemical expression of nNOS. **(B)** Expression pattern of GFP. **(C)** Overlay of **(A)** and **(B)**. nNOS-type I neurons display strong immunolabeling (open arrows) and a large soma whereas nNOS-type II (arrows) are weakly stained and display smaller soma. Note that all nNOS-positive neurons are GABAergic. Scale bar: 30 μm. Unpublished caption obtained from preparations used for the study presented by Perrenoud et al. (2012a).

In the cerebral cortex, nNOS GABAergic neurons comprise an average of 20% of the neocortical GABAergic population (Kubota et al., 1994; Gonchar and Burkhalter, 1997; Magno et al., 2012 and Perrenoud et al., 2012a in this issue). Classically, two types of GABAergic nNOS^+^ neurons have been distinguished at the histochemical level (Figure 2). The first one corresponds to the subpopulation of GABAergic neurons expressing high levels of nNOS and NADPH-d activity, the so called “nNOS-type I” that display fast-spiking and adapting properties. They account for 0.5–2% of the neocortical GABAergic population (Kubota et al., 1994; Gonchar and Burkhalter, 1997; Magno et al., 2012 and Perrenoud et al., 2012a in this issue). In these neurons nNOS is associated with SOM and NPY expression and immunoreactivity as well as with the substance P receptor NK1 (Kubota et al., 2011). Further, it was recently shown that these neurons are depolarized by substance P application (Dittrich et al., 2012 in this issue). They mainly correspond to projection neurons that are sparsely distributed in all neocortical layers but preferentially located in lower layer VI (Perrenoud et al., 2012a in this issue; Magno et al., 2012 in this issue) and to a lesser extent in superficial layers. Using NADPH-d activity these GABAergic neurons were recently shown to send long (>1.5 mm in the mouse) thick axonal fascicles running between the gray and white matter in cat and mouse neocortex invading both the corpus callosum and the fimbria (Tomioka et al., 2005; Higo et al., 2009). Their projections innervate both GABAergic neurons and pyramidal neurons and they are suspected to interconnect the two controlateral hemispheres as well as the archi- and paleo-cortex. Interestingly, nNOS-type I cells were recently shown to be selectively activated during sleep as they showed c-Fos accumulation during sleep recovery following sleep deprivation (Gerashchenko et al., 2008). Kilduff et al. proposed that nNOS-type I GABAergic neurons could synchronize EEG activity across neocortical regions (detailed in the last chapter; Kilduff et al., 2011).

**Figure 3:**
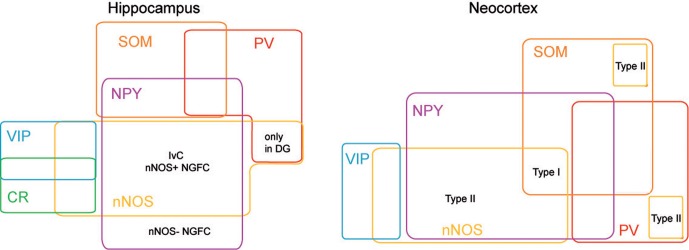
**nNOS expressing interneurons in cortex and hippocampus.** Scheme summarizing the molecular profiles of neocortical and hippocampal nNOS^+^ interneurons. This diagram is based on previous report (Tricoire et al., 2010, 2011) and on Perrenoud et al. (2012a in this issue).

The second classically defined subpopulation of neocortical nNOS expressing GABAergic neurons exhibits weak nNOS soma staining and low NADPH-d activity. This group corresponds to “nNOS-type II” cells that were initially reported in the primate (Yan et al., 1996; Smiley et al., 2000) but have more recently been described in rodents (Cho et al., 2010; Kubota et al., 2011). In rodents nNOS-type II GABAergic neurons comprise an average of 17% of the neocortical GABAergic population (Kubota et al., 2011; Magno et al., 2012 and Perrenoud et al., 2012a in this issue) and have often been underestimated due to the difficulty of their visualization. These cells mainly concentrate into the superficial layers II–III and in deep layers V–VI. Although poorly described “nNOS-type II” cells appear to form a heterogeneous cell population regarding the neuronal markers they co-express and the few electrophysiological properties that have been reported. Indeed, a fraction of “nNOS-type II” cells was reported to express SOM and another PV (Kubota et al., 2011; Vruwink et al., 2001) with both of these distinct subsets emerging clearly in the cluster analysis (Karagiannis et al., 2009). Another subpopulation of nNOS-type II neurons comprises the group of adapting neurogliaform interneurons that mediates slow GABAergic inhibition of pyramidal cells and interneurons (Karagiannis et al., 2009; Oláh et al., 2009). Indeed, in their classification of NPY^+^ interneurons Karragiannis and colleagues revealed that a fraction of interneurons expressing NPY^+^, but not PV, SOM, or VIP, and displaying adapting firing properties with neurogliaform morphologies could be further subdivided into two groups one expressing NPY “only” and another that accounts for 50% of the neurogliaform cluster in which NPY is co-expressed with nNOS (Karagiannis et al., 2009). In addition this cluster also included neurons expressing nNOS only but sharing electrophysiological and morphological similarities with adapting NPY interneurons.

More recently, Perrenoud et al performed a multiparametric analysis of “nNOS-type I” and “nNOS-type II” cells that intended to clarify nNOS expressing cell classification schemes and shed light on the physiological relevance of the different subgroups (Perrenoud et al., 2012a in this issue). This multiparametric analysis used an unsupervised classification of nNOS expressing GABAergic neurons and demonstrated clear segregation of nNOS cells into four clusters. One group contained GABAergic nNOS neurons co-expressing SOM and NPY that might correspond to the well-described population of nNOS-type I interneurons (Karagiannis et al., 2009; Kubota et al., 2011). Electrophysiologically these cells displayed adapting discharges fired long duration spikes followed by fast AHPs and had significantly slower membrane time constants than other interneurons. The three other clusters presumably corresponded to subpopulations of nNOS-type II interneurons. One cluster consisted of a population of interneurons co-expressing nNOS and CR and/or VIP that was to our knowledge not reported before. They were characterized by high input resistances, low firing threshold, adapting discharges to threshold and saturating current injections and they fired at significantly lower maximal frequencies than other neurons. A second cluster included a population of interneurons coexpressing nNOS and NPY with the exclusion of other classical markers (except CCK) that might correspond to neurogliaform interneurons. On an electrophysiological basis these NPY^+^/nNOS^+^ neurons were characterized by medium range input resistances. They displayed action potential discharges that were accelerating at threshold, adapting at saturation and a significantly larger accommodation of spike amplitude than in other clusters. In addition, these GABAergic neurons displayed long duration spikes followed by significantly slower AHPs than observed in other neurons. The third cluster included nNOS^+^ interneurons expressing PV or SOM that are mainly located in the infragranular layers. These neurons displayed several unique electrophysiological characteristics. They had depolarized membrane potentials and short time constants. Moreover, these cells showed little or no adaptation at threshold, fired at significantly higher maximal rates, and displayed significantly faster spike and AHP dynamics than other neurons.

## Development of telencephalic interneurons

**Figure 4:**
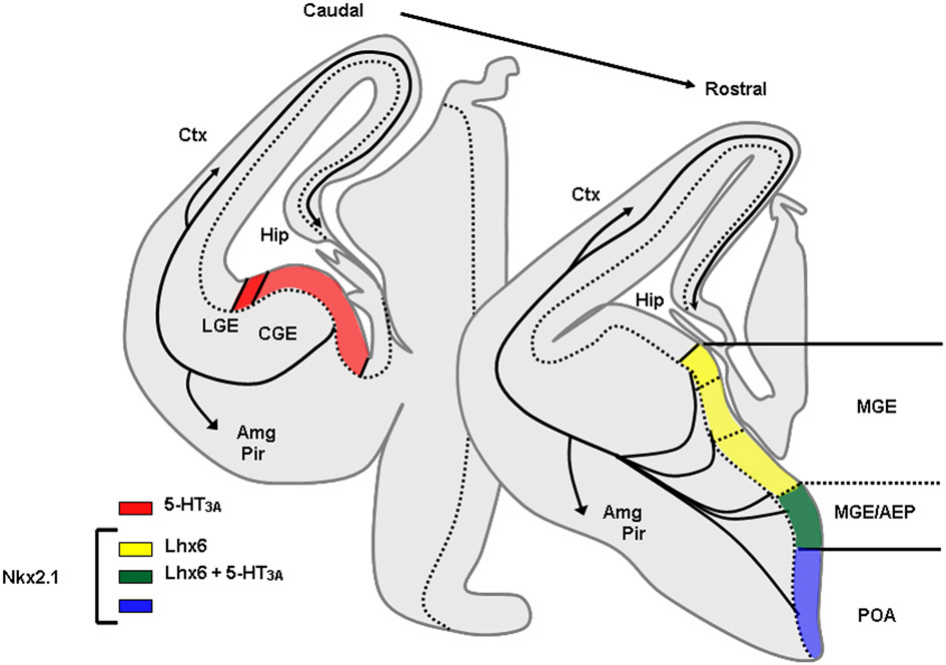
**Origins of GABAergic neurons populating the cerebral cortex and hippocampus at embryonic stages.** Transversal schematic sections of E13–E14 embryonic mouse brain showing regions relevant to origin/birth of cortical interneurons. Territories expressing specific transcription factors or molecules classically used to determine the place of genesis of specific interneurons subpopulation are drawn. AEP, entopeduncular region, Amg, amygdala, CGE, caudal ganglionic eminence; Ctx, cortex; Hip, hippocampus; LGE, lateral ganglionic eminence; MGE, medial ganglionic eminence; Pir, piriform cortex; POA, preoptic area.

**Figure 5:**
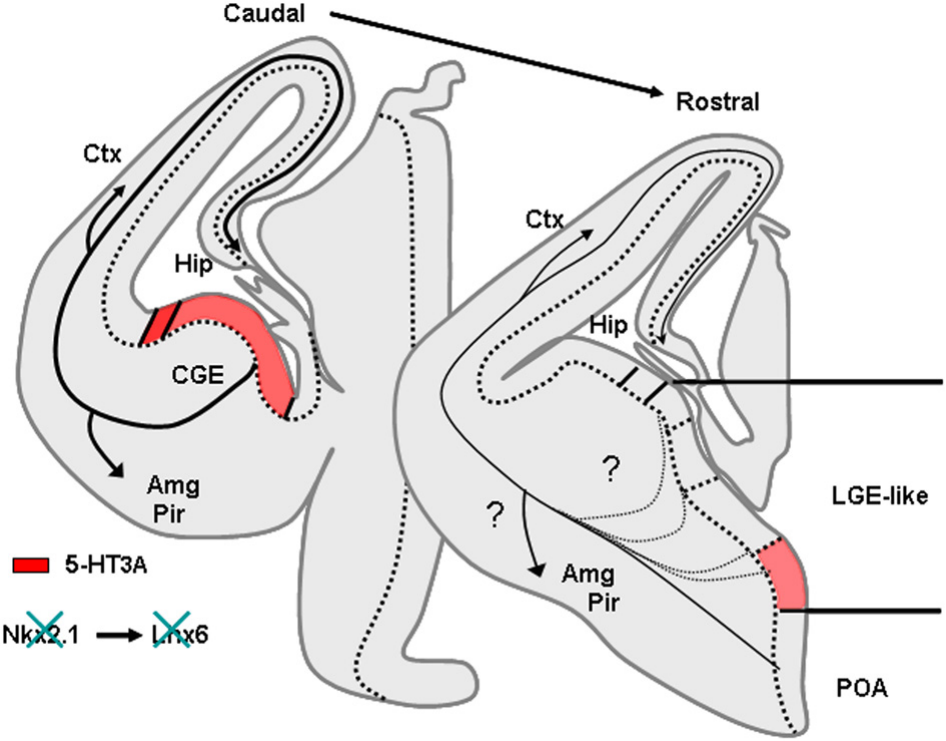
**Phenotype of mice lacking Nkx2.1.** Nkx2.1 knockout mice show a MGE respecified into a “LGE-like” territory. Since Nkx2.1 is necessary for Lhx6 expression, Lhx6 is not observed in these animals that lack most PV and SOM expressing neurons in the cortex and hippocampus. AEP, entopeduncular region, Amg, amygdala, CGE, caudal ganglionic eminence; Ctx, cortex; Hip, hippocampus; LGE, lateral ganglionic eminence; MGE, medial ganglionic eminence; Pir, piriform cortex; POA, preoptic area.

In rodents numerous studies have demonstrated that telencephalic interneurons mainly derive from subpallial territories (Figure 4). Pioneering *in vitro* studies and phenotypical descriptions of mutant mice lacking germinal zones that showed reduced interneuron numbers in the neocortex and hippocampus suggested that telencephalic interneurons expressing SOM and PV originate from the medial ganglionic eminence (MGE) and/or the preoptic area (POA) (Lavdas et al., 1999; Sussel et al., 1999; Wichterle et al., 1999; Pleasure et al., 2000; Wonders and Anderson, 2006; Batista-Brito and Fishell, 2009; Vitalis and Rossier, 2011). Indeed, in mice deficient for Nkx2.1, a transcription factor expressed in MGE and POA, the MGE appears to undergo a respecification into an LGE-like region and SOM and PV interneurons are dramatically reduced in the cortex and hippocampus. (Sussel et al., 1999; Pleasure et al., 2000; Figure 5). More recently, it was demonstrated that Nkx2.1 was necessary for the expression of Lhx6, a Lim homeobox transcription factor that is specifically expressed in the MGE and needed for the specification of MGE-derived interneurons (Liodis et al., 2007; Du et al., 2008). Grafting experiments and the use of transgenic mice often in association with “Cre-Lox strategy” have refined these analyses and confirmed that in the cerebral cortex fast spiking PV interneurons (Xu et al., 2004; Butt et al., 2005, 2007; Wonders et al., 2008) originate preferentially from the ventral part of the MGE (MGEv). By contrast, similar studies revealed that neocortical bursting and adapting SOM interneurons arise preferentially from the dorsal part of the MGE (MGEd) (Butt et al., 2005; Miyoshi et al., 2007). In the cerebral cortex, Martinotti cells co-expressing SOM and CR were further shown to be derived from the most dorsal MGE territory (LGE4 as named in Flames et al., 2007) that expresses the transcription factor Nkx6.2 (Fogarty et al., 2007). While initial *in vitro* experiments revealed that the CGE produces mainly CR expressing interneurons (Xu et al., 2004), more recent studies have demonstrated a much larger contribution of this region in generating telencephalic interneuron diversity (Butt et al., 2005; Fogarty et al., 2007; Miyoshi et al., 2007; Lee et al., 2010; Vucurovic et al., 2010). Indeed, together these studies showed that telencephalic (hippocampal and neocortical) interneurons expressing VIP, CR, and a subpopulation of neocortical neurogliaform interneurons expressing NPY (Lee et al., 2010; Tricoire et al., 2010, 2011; Vucurovic et al., 2010) are all CGE-derived. Interneurons arising from CGE pogenitors all appear to express the 5-HT receptor type 3A (5-HT_3A_) (Lee et al., 2010; Vucurovic et al., 2010) and the transcription factor Gsh2 (Fogarty et al., 2007) while lacking Nkx2.1, Nkx6.2, and Lhx6 (Flames et al., 2007). However, it should be noted that the entopeduncular region (AEP), also defined as the more ventral extension of the MGE (Flames et al., 2007) co-expresses 5-HT_3A_, NKx2.1, and Lhx6. Homochronic grafting of the AEP has revealed that this region does not appear to contribute importantly to the genesis of neocortical neurons expressing 5-HT_3A_. By contrast, these experiments have shown that the AEP generates subpopulations of 5-HT^+^_3A_ hippocampal interneurons (Vucurovic et al., 2010; Jaglin et al., 2012).

Besides contributions from the MGE, CGE, and AEP other regions have been implicated in the genesis of neocortical and hippocampal interneurons such as the preoptic regions and the neocortex. Recently, homochronic graftings of dorsal preoptic territories (POA1) have revealed that Nkx5.1^+^ progenitors generate neocortical interneurons expressing NPY^+^ with the exclusion of other markers classically used to discriminate interneurons populations (Gelman et al., 2009). The anatomical features and firing patterns of these neurons in the neocortex suggested they represent an additional subset of neurogliaform interneurons (Gelman et al., 2009). Further, Gelman et al have shown that the Dbx1-derived progenitors arising from the ventral POA (POA2) contribute to the genesis of various interneurons including fast spiking PV^+^, SOM^+^, multipolar late spiking NOS^+^, neurogliaform, and bituftued/bipolar irregular spiking VIP/CR interneurons that mainly populate deep neocortical layers and hippocampal subfield (Gelman et al., 2011).

Together these studies have successfully correlated the place of genesis and the contribution of specific transcription factors or molecular markers with a preferential interneuron phenotype and location. Specific guidance molecules are preferentially expressed in different subterritories and participate to the targeting of specific interneuron subpopulations. Recent studies suggest that motility and guidance of interneurons depend on several molecular cues that are already differentially expressed in ganglionic eminences and neocortical compartments (Powell et al., 2001; Polleux et al., 2002; Pozas and Ibanez, 2005; Kanatani et al., 2008; López-Bendito et al., 2008). However, other mechanisms have been shown to participate in the correct positioning of specific classes of interneurons. Indeed, the selective cell death of specific interneurons during early postnatal development may contribute to remove those that are abnormally positioned or not appropriately integrated in neocortical circuits (De Marco García et al., 2011). For instance it has recently been shown that reelin^+^ and CR^+^, but not VIP^+^, interneurons depend on neocortical activity for their correct migration and positioning (De Marco García et al., 2011).

In addition to the embryonic genesis of neocortical interneurons recent studies have also shown that during the three first postnatal weeks the neocortex produces CR-positive interneurons (Cameron and Dayer, 2008; Inta et al., 2008; Riccio et al., 2012). Such postnatally generated populations may participate in distinct physiological processes including the appropriate targeting of callosal projections.

## Origin of interneurons expressing nNOS

### Origin of hippocampal interneurons expressing nNOS

**Figure 6:**
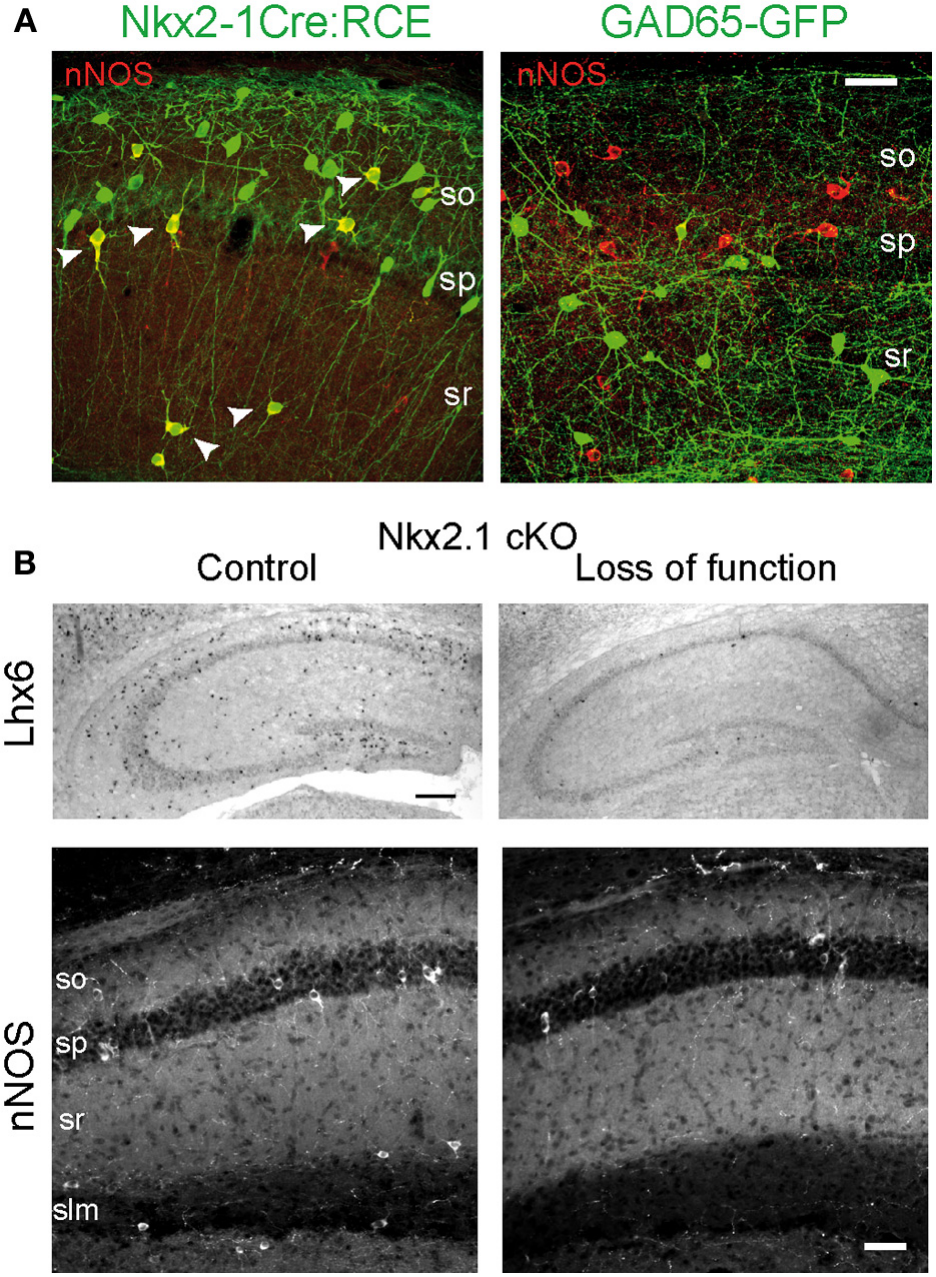
**Embryonic origin of nNOS^+^ hippocampal interneurons. (A)** Images illustrating the coexpression of GFP and nNOS in the Nkx2.1Cre:RCE (left) and GAD65-GFP (right) mouse lines. Scale bar: 25 μm. **(B)** Nkx2.1 is necessary for the specification of nNOS^+^ interneurons. Top, *In situ* hybridization against Lhx6 transcripts on hippocampus of control (left) and mutant (right) P15 mice after conditional loss of Nkx2.1 function at E10.5. Scale bar: 200 μm. Bottom, Immunohistochemical expression patterns of nNOS in CA1 of control and mutant mice. Scale bar: 50 μm. Adapted from Tricoire et al. (2010, 2011).

As mentioned above, hippocampal nNOS^+^ interneurons differ from their neocortical homologs in terms of neuronal diversity and distribution among hippocampal subfields and layers. Therefore specific studies have addressed their embryonic origin using lineage analysis, conditional fate-mapping, and loss of function (Fogarty et al., 2007; Tricoire et al., 2010, 2011; Figure 6). Using an Nkx2.1-Cre driver line in combination with different Cre-dependant GFP reporter lines, it has been shown that IvCs and nNOS^+^/NGFCs derive essentially from the MGE. This was also supported by the expression of Lhx6 in these subpopulations (Tricoire et al., 2011; Figure 6). Accordingly, when a CGE preferred tamoxifen dependant driver line was used (Mash1-CreER, Miyoshi et al., 2010), very few fate-mapped neurons expressed nNOS (Tricoire et al., 2010). These few nNOS expressing CGE-derived neurons typically exhibited morphologies and distributions consistent with VIP^+^/CR^+^ interneurons rather than IvCs and NGFCs. Moreover, conditional loss of Nkx2.1 function (constitutive knock out die at birth) caused an almost complete loss of nNOS^+^ GABAergic neurons in the hippocampus except for few bipolar interneurons in s.p. reminiscent of the VIP^+^/CR^+^ interneurons revealed in the CGE reporter (Tricoire et al., 2010; Figures 6A,B). In parallel, analysis of a GAD65-GFP transgenic line that labels a subset of CGE-derived interneurons (López-Bendito et al., 2004) further confirmed that some VIP^+^ interneurons also express nNOS. Their electrophysiological and morphological properties were different from those of IvCs and NGFCs but were reminiscent of the IS-3 cell type that inhibits a subset of SOM^+^ interneurons located in stratum oriens (s.o.) that project in turn to the stratum lacunosum molecular (Freund and Buzsáki, 1996; Chamberland et al., 2010).

Surprisingly, the lineage analysis also revealed that classically defined NGFCs can be subdivided into two groups with nNOS^+^/NGFCs being derived from the MGE and nNOS-/NGFCs arising from CGE progenitors (Tricoire et al., 2010, 2011). This contrasts with findings in the neocortex where the CGE is the dominant source of NGFCs (Butt et al., 2005; Miyoshi et al., 2007, 2010) and of nNOS-type II interneurons (Perrenoud et al., 2012a in this issue). The surprising lack of nNOS in the CGE-derived subset of NGFCs may partially explain the reduced levels of nNOS in the neocortex compared to the hippocampus (Yan and Garey, 1997; Lee and Jeon, 2005). The striking difference between hippocampal and neocortical NGFCs suggests that interneuron precursors could be fated early during embryogenesis to reside in either the hippocampus or neocortex, perhaps reflecting differential sensitivities to specific sorting factors like chemokines (Li et al., 2008; López-Bendito et al., 2008) that promote migration of nNOS^+^/NGFC and IvC precursors into the hippocampus. Alternatively, these cells may adopt a different fate depending on whether they integrate into the hippocampus or neocortex due to differential expression of morphogenic molecules within these local environments.

### Origin of neocortical interneurons expressing nNOS

Investigations into the developmental origins of neocortical GABAergic neurons expressing nNOS are only in their infancy due to the fact that this population in the juvenile brain is largely heterogeneous and thus poorly defined. This is especially true for nNOS-type II interneurons that display low NADPH-d activity and nNOS-immunoreactivity making them difficult to identify histologically. The study presented by Perrenoud et al. in this special issue is to our knowledge the first study to specifically characterize neocortical interneurons expressing nNOS using a multiparametric approach and to elucidate their developmental origins (see Table 1). The first group identified is homologous to previously described nNOS-type I cells being relatively homogeneous comprised of nNOS^+^ GABAergic cells that coexpress SOM and display fast-spiking properties (Perrenoud et al., 2012a in this issue; see above). These properties clearly suggest that they belong to a subgroup of well-defined SOM^+^ interneurons that were previously shown to derive from the MGE. Indeed, Perrenoud et al. demonstrate that all members of this subgroup express Lhx6 in agreement with two recent studies—presented in this issue—that have used various transgenic mouse lines to clarify the origin of nNOS expressing interneurons (Jaglin et al., 2012; Magno et al., 2012). Interestingly, it was recently shown that the specification of a large fraction of nNOS-type I neurons required the Lhx6-mediated activation of Sox6 for proper specification (Batista-Brito et al., 2009; Jaglin et al., 2012). Indeed, deletion of Sox6 in Lhx6 expressing cells suppressed SOM expression in nNOS-type-I neurons and altered their morphology by decreasing process complexity (Jaglin et al., 2012). In contrast to this first cluster, nNOS-type II cells displayed considerable heterogeneity segregating into three clusters with embryonic origins in both the MGE and the CGE/AEP territories. Indeed, not all nNOS-type II cells express Lhx6 (Jaglin et al., 2012 in this issue; Perrenoud et al., 2012a). A subpopulation of nNOS-type II cells express 5-HT_3A_, a CGE/AEP marker, and colocalization between nNOS, 5-HT_3A_, and VIP was observed (average 10% of the 5-HT_3A_ population; Perrenoud et al., 2012a,b in this issue). These cells are mainly localized in the superficial layers where they may participate in neuro-vascular coupling. Another group of cells expressing nNOS and NPY but not SOM may derived from MGE and CGE territories and could correspond to neurogliaform cells located in the most superficial layers where they may bidirectionally regulate blood flow. The recent genesis of a transgenic line expressing a tamoxifen inducible Cre recombinase under the control of the nNOS promoter (nNOS-CreER) will help to analyze the physiological roles that these populations may play (Taniguchi et al., 2011).

### Primates and human telencephalon: specific aspects of GABAergic development

Rodents, specifically mice, are of great interest due to the availability of transgenic models (Taniguchi et al., 2011) that allow for thorough dissection of the genetic programs needed for interneuron development and specification. However, it is difficult to relate neocortical development in mice to the much longer timescale and complexity of primate development (Uylings et al., 1990; Rakic, 2009). Indeed, comparative studies across species indicate that the first postnatal week in mice corresponds broadly to gestational days 85–130 in macaques and to 110–170 in humans (Clancy et al., 2001). The much longer timescale in these higher order species is certainly due to the important brain expansion in size and therefore to the increasing distance of subpalial and pallial territories and concerns the place of origins of telencephalic interneurons (see Molnar et al., 2006; Rakic, 2009). Indeed, while the vast majority of telencephalic GABAergic neurons originate from supallial territories in rodents (see above), in humans (from 5 to 15 gestational weeks) and primates this is only the case for the first generated ones that mainly arise from MGE (Letinic et al., 2002; Jakovcevski et al., 2011; Zecevic et al., 2011). Later, neurogenesis occurs in dorsal pallial territories and presumably in the CGE (Petanjek et al., 2009a,b; Jakovcevski et al., 2011). Indeed, it is known that late proliferations from pallial territories mainly generate CR^+^ interneurons that are more numerous in humans and primates than in rodents and display distinct morphologies in each species (Jones, 2009; Rakic, 2009).

Recently, analysis of interneuron densities in postmortem brain tissue from humans suffering from holoprosencephaly associated with agenesis of GE showed a strong correlation between massive reductions in Nkx2.1 expression and depletion of nNOS/NPY/SST^+^ and PV^+^ interneurons (Fertuzinhos et al., 2009). These observations suggest that, like in mice, these populations of putative nNOS-type I cells are generated in the GE. Despite the fact that nNOS-type II largely outnumbered nNOS-type I neurons in primate and human brains their place of genesis has not been analyzed in these species.

## Development and maturation of neocortical and hippocampal interneurons expressing nNOS

The pattern of nNOS immunoreactivity in the rodent telencephalon undergoes sterotyped changes during development. From embryonic day 13 (E13) to the first postnatal day (P0), a period of intense neuronal migration, nNOS is strikingly expressed by distinct cells types. Indeed, cells migrating in the marginal zone displaying Cajal-Rezius like morphologies express nNOS (Santacana et al., 1998). In addition, by E15 in rats, nNOS labeling is clearly seen in the ganglionic eminence and the AEP/PO region (Figure 2A in Santacana et al., 1998) suggesting that nNOS could also label the early populations of GABAergic neurons that continue to express nNOS at mature stages. Later on, from E17 to E19, in rats, neurons displaying leading processes oriented along the intermediate zone or toward the pial surface, presumably migrating neurons were reported to express nNOS (Santacana et al., 1998). However, it is not clear whether they correspond solely to GABAergic neurons or to subpopulations of GABAergic and glutamatergic neurons.

In rat visual cortex, nNOS^+^ neurons appear as early as postnatal day 1 in the intermediate (white matter) and subplate (layers V and VI) regions as small and undifferentiated neurons. Differences in intensity of nNOS immunoreactivity (later mentioned as type I and type II neurons) become evident as early as P7 (Chung et al., 2004; Kanold and Luhmann, 2010). nNOS GABAergic neurons reach their typical morphology in the second postnatal week and appear in all layers. Neurons in layers V and VI precede those in the superficial layers in acquiring their final morphology. By P30, NADPH-d active neurons are no longer detected in layer I suggesting they die off or migrate to deeper layers residing only transiently in layer I (Lüth et al., 1995).

In rat barrel cortex, an area that integrates sensory inputs coming from the whiskers, between P10 and P90, the neuropilic distributions of NADPH-d and cytochrome oxidase (CO) activities exhibit a remarkable similarity. NADPH-d activity is denser in barrel hollows, regions that receive somatotopic sensory thalamic inputs, and is less active in barrel septa (Furuta et al., 2009). The number of NADPH-d active neurons increases significantly in the barrel fields between P10 and P23, with a peak at P23. The dendritic arborizations of NADPH-d active neurons become more elaborate during barrel development. At all ages evaluated, the number of NADPH-d^+^/NOS^+^ cells, mostly type I cells, was always higher in the septa than in the barrel hollows (Vercelli et al., 1999; Freire et al., 2004).

In the hippocampus, nNOS is transiently expressed in the pyramidal cell layer between P3 and P7 (Chung et al., 2004). While NADPH-d reactive soma and processes are present from the day of birth until adulthood in Ammon's horn, expression of NOS is delayed in the DG appearing only by the end of the first postnatal week (Moritz et al., 1999).

## Role of nNOS and NO in development, maturation, and plasticity

### Production of nitric oxide by neuronal nitric oxide synthase

**Figure 7:**
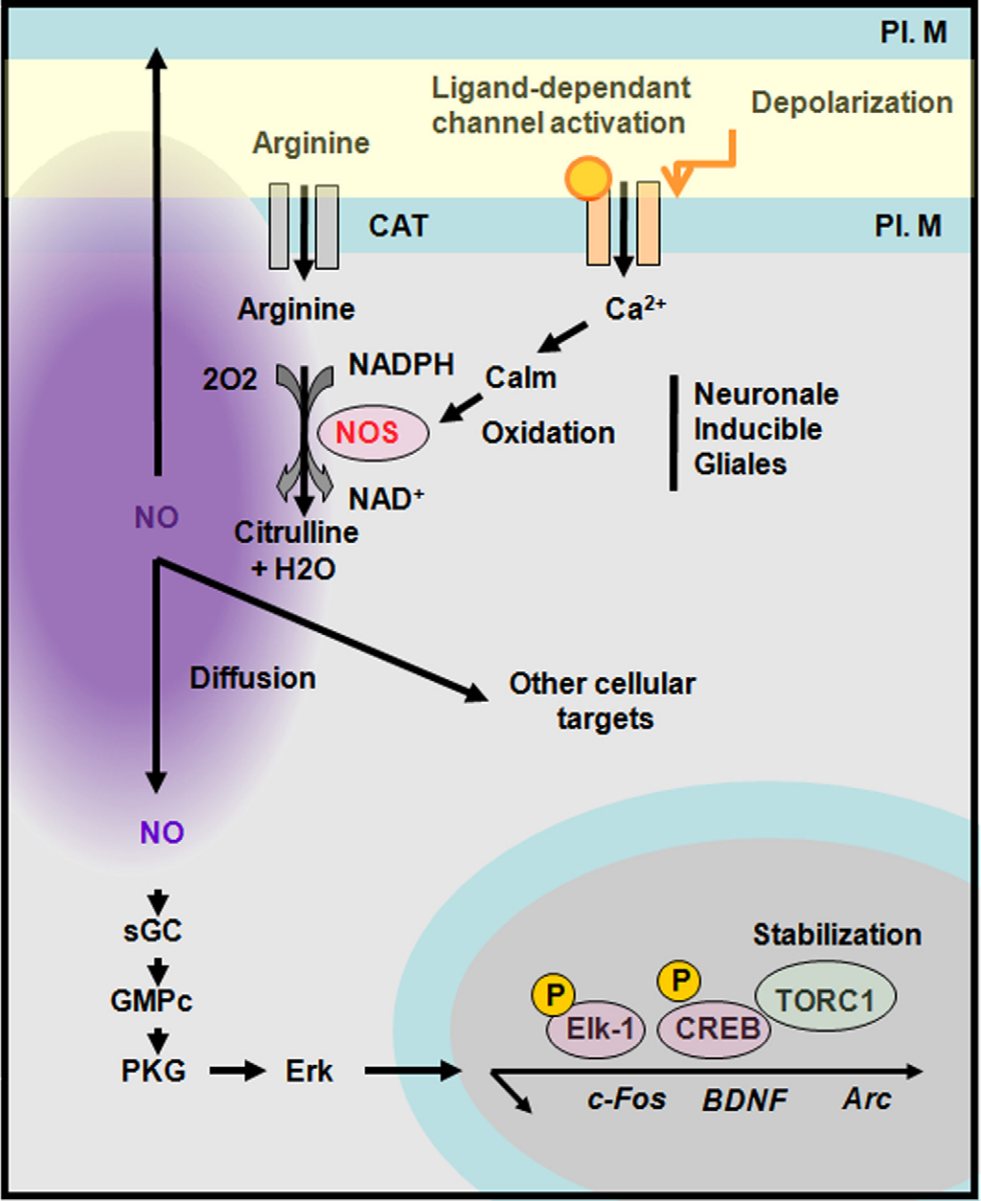
**Synthesis of nitric oxide and transduction cascades.** Neuronal nitric oxide synthase (nNOS) is activated by a calcium-dependant calmodulin. NOS produces nitric oxide (NO) upon oxidation of arginine into citrulline. NO diffuses and act on presynaptic or postsynaptic targets. A well-known pathway of NO is through the activation of guanylyl cyclase (GC) that activates a protein kinase G (PKG) leading to Erk activation and the stabilization of TORC1 a CREB co-activator. CAT, cation and anion transporter; PL. M, plasma membrane. Adapted from Gallo and Iadecola (2011).

NO is a free radical gas that can move rapidly across plasma membranes in anterograde and retrograde directions to act presynaptically, postsynaptically or within the cell that has produced it. NO is generated following the activation of NO synthases (Bredt and Synder, 1990; Daff, 2003). So far three NOS isoforms have been identified, two of which, endothelial (eNOS) and the neuronal (nNOS), are constitutively expressed while the third one is inducible and rarely present under basal conditions. Each NOS subtype has distinct functional and structural features. Depending on the neuronal cell type and the mode of neuronal excitation, nNOS, which is a Ca2^+^/calmodulin-regulated enzyme, can be activated by Ca^2+^ influx through N-methyl-D-aspartate (NMDA) receptors or other calcium permeable channels (i.e., the ionotropic 5-HT_3A_ receptor; Rancillac et al., 2006; see also Perrenoud et al., 2012b in this issue). Alternatively, calcium liberated from intracellular stores such as the endoplasmic reticulum (i.e., through activation of metabotropic receptors coupled to activation of Gq protein) may promote nNOS activity. Arginine transported into the cell by the anion-cation tranporter is oxidised by nNOS into citrulline in a nicotinamide adenine dinucleothide phosphate (NADPH)-dependant manner (Snyder et al., 1998) generating NO that is considered to be stable in physiological conditions for approximately 1–2 s (1 s half-life; Garthwaite, 2008). Within cells, NO has the capacity to trigger several transduction pathways. The most well-known involves activation of guanylyl cyclase (Arnold et al., 1997) leading to the conversion of GTP into cGMP and subsequent activation of protein kinase G (PKG). PKG activity in turn promotes Erk activation and the induction of various immediate early genes such as c-fos, Arc, and BDNF. Indeed, in neuronal cultures NOS inhibition attenuates bicuculine-induced activation of Erk as well as the rise in c-Fos, Egr-1, and Arc that are all implicated in experience-dependant plasticity in the barrel cortex. Moreover, although NOS inhibition does not affect the phosphorylation of CREB it decreases accumulation of the CREB coactivator TORC1 (Gallo and Iadecola, 2011; Figure 7). Activation of the NO/cGMP pathway is implicated in various neurophysiological processes including neuronal development, synaptic modulation, learning and memory. In addition several cGMP-independent effects of NO related to nervous system function have been reported. For instance, various presynaptic targets for NO have been identified such as SNAP25, synthaxin Ia, n-Sec 1, neurogranin as well as the postsynaptic targets ADP ribosyltransferase and NMDA receptors (Gallo and Iadecola, 2011). Finally, excessive NO production is potentially neurotoxic but this aspect is beyond the scope of this revue (Steinert et al., 2010).

### Role of nNOS and NO during early development

Numerous papers and reviews have described the role of nNOS and NO in various neuronal populations during development. Here, we will briefly focus on some of the best understood roles for NO/nNOS in neurons at early stages. nNOS or NADPH-d activity are transiently expressed in the embryonic hippocampal and neocortical anlagen during the peak of neurogenesis and the period of developmental synaptogenesis (Bredt and Snyder, 1994). It has been shown that NO acts as a paracrine messenger in newly generated neurons to control the proliferation and differentiation of mouse brain neural progenitor cells (NPC). Treatments with the NO synthase inhibitor L-NAME or the NO scavenger hemoglobin increase cell proliferation and decrease the differentiation of NPCs into neurons (Barnabé-Heider and Miller, 2003). Interestingly, a similar role of NO was demonstrated in the subventricular zone of adult mice, a region that retains the capacity to generate neurons at mature stages (Xiong et al., 1999; Cheng et al., 2003; Matarredona et al., 2004). Both BDNF and epidermal growth factor (EGF) have been largely implicated in these events (Barnabé-Heider and Miller, 2003; Matarredona et al., 2004).

In addition to regulating neurogenesis, NO has also been implicated in the formation of cerebral maps. This role has been largely investigated and demonstrated in the visual system where NO induces synaptic refinement or elimination of immature synaptic connections at retino-collicular and retino-thalamic levels (Cramer et al., 1995, 1996; Wu et al., 1996, 2000; Cramer and Sur, 1999; Cuderio and Rivadulla, 1999; Vercelli et al., 2000). However, outside of retino-collicular and retino-thalamic organization, NO appears dispensible for the establishment of patterned neocortical maps since animals receiving daily injection of nitroarginine prior to and during the period of ocular dominance column formation, as well as nNOS knockout mice, display normal organization of the somatosensory cortex and barrel field plasticity (Van der Loos and Woolsey, 1973; Finney and Shatz, 1998). Nevertheless, though apparently not instructive, NO may still participate in establishing and refining neocortical connectivity. Indeed, when NADPH-d activity is altered in the barrel field, as observed in mice lacking NMDAR1 specifically in neocortical neurons, abnormal segregation of thalamocortical axons occurs (Iwasato et al., 2000; Lee et al., 2005). In these animals thalamocortical axons display fewer branch points in layer IV and abnormally expansive thalamocortical arbors, a feature that corresponds to a rudimentary whisker-specific pattern. These results suggest that NO could promote thalamocortical sprouting and participates in the consolidation of synaptic strength in layer IV of the primary somatosensory cortex.

Finally, it has been shown that between P6 and P10 in rodents, NO also affects neuronal gap-junction coupling. Indeed, Rörig and colleagues have shown that following preincubation with sodium nitroprusside (an NO donor), the number of gap-junction coupled neurons decreased (Rörig and Sutor, 1996a,b; Roerig and Feller, 2000). In the developing neocortex, gap-junctions represent a transient metabolic and electrical communication system occurring between glutamatergic or GABAergic neurons belonging to the same radial column. Thus, NO mediated regulation of gap junctions has the capacity to affect electrical coupling, synchronization of metabolic states and, coordination of transcriptional activity amongst connected neurons.

### Role of nNOS and NO in microcircuits plasticity

The idea that NO might modulate synaptic transmission, first proposed in 1988 by Garthwaite and colleagues (Garthwaite et al., 1988), has been confirmed in several brain regions including the hippocampus, striatum, hypothalamus, and locus coeruleus (Prast and Philippu, 2001). Indeed, studies using NO donors suggest that release of several transmitters, including acetylcholine, catecholamines, glutamate and GABA are regulated by endogenous NO. As a gaseous very weakly polar molecule without net electric charge and due to its small size, NO can diffuse readily across cell membranes. However, the high reactivity of NO as a free radical limits activity to within a micrometer of its site of synthesis allowing for synapse specificity in modulating presynaptic function (Garthwaite, 2008).

In acute hippocampal slices from neonatal rat, NO signaling was found to decrease GABAergic and glutamatergic postsynaptic currents, whereas network calcium imaging indicated that inhibition or stimulation of NO signaling enhanced or suppressed synchronous network events, respectively (Cserép et al., 2011). The regulation of GABAergic and glutamatergic synaptic transmission in early postnatal development, NO is considered particulalrly critical for fine-tuning synchronous network activity in the developing hippocampus (Cserép et al., 2011). In more mature hippocampus NO regulates LTP at the Schaffer collateral/CA1 synapses and acts as a retrograde messenger (for review see Malenka and Bear, 2004; Lisman and Raghavachari, 2006). This occurs via the activation of postsynaptic NMDA receptors, synthesis of NO by NOS expressed in pyramidal cells and then retrograde activation of guanylate cyclase located in axon terminals (See Feil and Kleppisch, 2008 for detailed intracellular mechanisms). In contrast, in the cerebellum NO serves as an anterograde messenger that is produced in parallel fiber terminals or cerebellar interneurons and then diffuses to the postsynaptic Purkinje cell to induce LTD through a cGMP-dependent mechanism (for review see Feil et al., 2005).

### Role of NO and interneurons expressing nNOS in hippocampal and neocortical network

Studies investigating synaptic modulation by NO have typically considered it to be derived from NOS localized in pyramidal cell postsynaptic densities. However, as described above, nNOS is largely expressed in GABAergic interneurons. Even if NO can modulate GABAergic transmission, it is still unclear if the NO released by interneurons principally regulates transmitter release or instead participates in other homeostatic processes such as regulation blood flow or neuronal excitability (Iadecola et al., 1993). Indeed bath application of an NO donor onto acute rat neocortical slices cause dilation of blood vessels (Cauli et al., 2004) and this hemodynamic change can similarly be elicited electrical stimulation of a single neocortical nNOS expressing interneuron (Cauli et al., 2004). Such tight coupling between neuronal activity of interneurons expressing nNOS and vasomotricity has also been reported in other brain structures such as cerebellum where pharmacological or electrical stimulation of stellate cells, which strongly express nNOS, induces vasodilation by release of NO that can be measured using NO-sensitive electrode (Rancillac et al., 2006). Given this interneuron mediated regulation of brain blood perfusion, it is interesting to note that most of nNOS^+^ interneurons also coexpress NPY which is a potent vasoconstrictor (Dacey et al., 1988; Cauli et al., 2004). Consistently, we have shown that activation of serotonin type 3 receptors which are present on nNOS-type II interneurons co-expressing NPY and/or VIP (Vucurovic et al., 2010; Perrenoud et al., 2012a in this issue), induces both vasodilation and vasoconstriction (Perrenoud et al., 2012b in this issue) via direct release of NO and NPY respectively. Therefore, it appears that both neocortical and hippocampal NGFCs, which coexpress NPY and nNOS, likely exert dual control over cerebral blood flow. To resolve these conflicting observations we propose that NPY, which is likely released at axon terminals, controls blood vessel tone distally from the cell body while NO released by the somato-dendritic compartment acts more proximally via volume transmission. These differential effects would permit fine-tuning of energy and oxygen supply by creating locally a microsphere with increased blood perfusion consequently to increased neural activity (Estrada and DeFelipe, 1998).

Regarding excitability, NO can regulate several conductances via the cGMP/PGK pathway in central neurons (Garthwaite, 2008). Indeed the hyperpolarization activated current that serves as a pacemaker to generate rhythmic activity amongst thalamic neurons (Pape and Mager, 1992) is regulated by NO (Biel et al., 2009). NO also acts on several potassium conductances such as the delayed rectifier Kv3 channels (Rudy and McBain, 2001). It has been shown that NO donors inhibit both Kv3.1 and Kv3.2 channels in CHO cells via activation of the cGMP/PKG pathway (Moreno et al., 2001). Such inhibition of Kv3 current has also been observed in the central nervous system via volume transmission in the auditory brain stem and the hippocampus (Steinert et al., 2008, 2011). It is interesting to note that Kv3 channels are responsible for the short duration of action potentials in auditory neurons as well as in hippocampal/neocortical PV^+^ and SOM^+^ interneurons (Atzori et al., 2000; Tansey et al., 2002; Lien and Jonas, 2003). NO-mediated modulation of Kv3 would therefore regulate the spike timing of these neurons (Lien and Jonas, 2003).

Recently, the role of NO in sleep regulation has been challenged. Indeed, the group of Kilduff has shown that long range projecting nNOS-type I GABAergic neurons are specifically activated during sleep by demonstrating that these cells specifically accumulate c-Fos during sleep rebound following sleep deprivation (Gerashchenko et al., 2008). The mechanism behind this activation is not completely understood. However, it is suspected that during the waking period NPY^+^/SOM^+^/nNOS^+^ GABAergic neurons (putative nNOS-type I) are inhibited by neuromodulatory afferents driving arousal such as acetylcholine, noradrenaline, serotonin, and histamine and that they would be activated when arousal systems are depressed when sleep-promoting substances are released (i.e., adenosine, cytokines, growth hormone, releasing hormone, and cortistatin). Once activated nNOS neurons could synchronize EEG activity across neocortical regions through the release of NO, GABA or NPY. Interestingly it has been reported that nNOS knockout mice spend more time than controls in slow wave sleep as monitored by EEG. This suggests that nNOS-type I GABAergic neurons may regulate sleep homeostasis (Kilduff et al., 2011). However additional experiments remain to be performed to fully address this point.

## Conclusion and perspectives

The development and plasticity of nNOS^+^ interneurons needs to be confronted with more general questions that are central to understand interneurons development and specification. One important issue to address is to determine the extent to which interneurons are fully specified by their place and time of genesis. In other words are these cells hard wired from the progenitor stage or allowed a certain degree of “developmental plasticity” after the last division of the progenitors to adapt to their migratory and ultimately circuit environment? At mature stages interneuron subtypes are characterized by a combination of: (1) their laminar position within different circuits; (2) specific combinations of neurochemical markers; (3) their basic morphology; and (4) their electrophysiological features including passive membrane properties, spiking behavior and synaptic connectivity. Various studies including some highlighted above have shown that these criteria are largely dictated by an interneuron's site and time of genesis. However, some studies have also pointed to a role for the cellular environment an interneuron ultimately occupies in refining these properties such as their stratification (i.e., CR- and reelin-positive interneurons) and their expression of certain activity regulated markers like NPY. In this respect it should be mentioned that the expression of nNOS appears to be developmentally regulated in various neuronal populations and could be modulated by cellular targets in subpopulations of interneurons (i.e., in an activity-dependent manner). Thus, although challenging, it will be important to determine whether nNOS interneurons are guided to their final location early on, like most interneurons, or are eliminated if inaccurately positioned or if they stop expressing/fail to induce nNOS. An understanding of subtle differences in the genetic makeup/molecular characteristics of divergent nNOS interneuron cohorts may provide insight into these issues. The recent generation and use of Cre reporter animals in association with other techniques have been successfully used to determine the embryonic origin and birthdating of nNOS type I and type II interneurons revealing for the first time their heterogeneity and specificities (lineage and characteristics displayed at mature stage; in this issue). The increasing array of transgenic models and genetic tools available (i.e., optogenetic) will help advance the pace of this research.

Interestingly, the unique features that have been shown to depend on neuronal activity (Verhage et al., 2000) for wiring and plasticity are the density and strength of GABAergic innervations. It remains to be established if and how NO could participate in the maturation and refinement of axonal and/or dendritic arborization of specific classes of interneurons.

### Conflict of interest statement

The authors declare that the research was conducted in the absence of any commercial or financial relationships that could be construed as a potential conflict of interest.
